# Scaphoid Fracture in a Patient with a Scaphotrapezial Synostosis: A Case Report and Literature Review

**DOI:** 10.1155/2017/1941750

**Published:** 2017-01-18

**Authors:** Soliman Noureldin, Mohammed Ali, Farshid Fallahi, Thomas Dehler

**Affiliations:** North Cumbria University Hospitals NHS Trust, Cumbria, UK

## Abstract

*Introduction.* Scaphotrapezial synostosis has been rarely reported in the literature and only one case underwent surgical treatment for scaphoid fracture.* Presentation of Case.* A 15-year-old male presented with a painful left wrist following a fall. The initial radiographs showed a displaced scaphoid proximal pole fracture and a Scaphotrapezial synostosis. The fracture was then fixed percutaneously with satisfactory outcome.* Discussion.* Scaphotrapezial synostoses are very rare and most found in patients with multiple congenital anomalies or as part of a hereditary syndrome. They have previously been reported; however, we found only one case reporting a concomitant scaphoid fracture.* Conclusion.* This is the second case of its kind to report surgical treatment of scaphoid fracture associated with a congenital Scaphotrapezial synostosis.

## 1. Introduction

Carpal synostosis is a rare anatomical variant that has been defined as an intrauterine failure of incomplete cavitation of the common cartilaginous precursors [1]. It is present in approximately 0.1% of the population with high incidence in females and people of African descent. These coalitions are invariably asymptomatic but can be cumbersome following trauma as reported by Simmons and McKenzie and DeFazio et al. [2, 3].

## 2. Case Report

A 15-year-old right handed boy of Caucasian origin presented with pain in the left wrist following a fall onto the outstretched hand. Clinical examination of the hand and wrist revealed tenderness in the anatomical snuff box with a positive axial thumb loading test.

**Figure 1:**
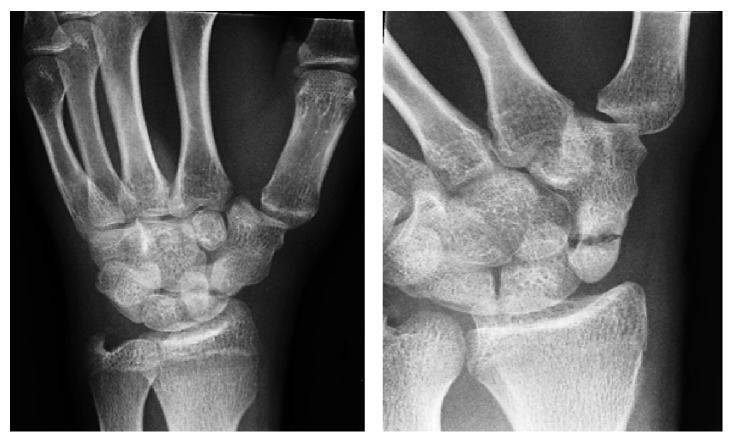
Radiographs show a displaced fracture through the waist of the scaphoid with Scaphotrapezial synostosis.

Radiographic examination of left wrist and hand confirmed a complete fracture through the proximal pole of the scaphoid and an associated fracture of the tip of the ulnar styloid process. Radiographs also showed a concomitant Scaphotrapezial synostosis (Figure 1).

**Figure 2:**
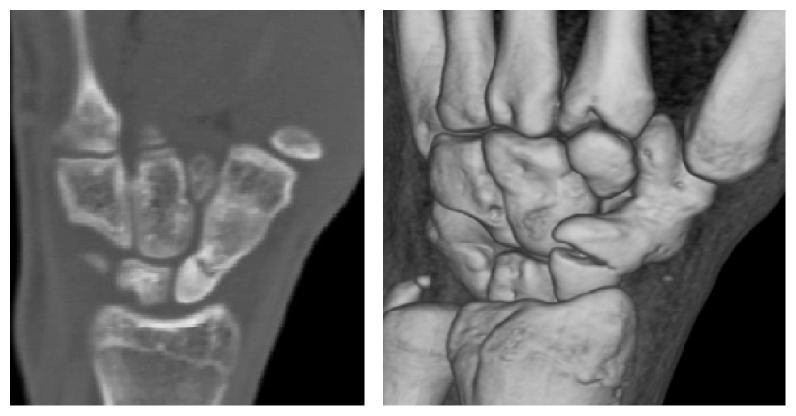
CT scan shows complete Scaphotrapezial synostosis with scaphoid waist fracture.

Patient's wrist was then placed in a below elbow back-slab and referred for an urgent CT scan for further detailed imaging and to help planning management. CT scan showed a fracture gap measuring about 2.5 mm, mild dorsal tilt of the lunate, and complete Scaphotrapezial synostosis (Figure 2).

Patient underwent antegrade percutaneous fixation of the scaphoid fracture via a dorsal approach using a headless, self-tapping, and variable-pitch compression screw and placed in a scaphoid cast.

Patient was then reviewed at six weeks with radiographic examination and because there was not enough evidence of healing, the cast immobilisation period was extended to four more weeks. Ten weeks postoperatively, the radiographic appearances showed evidence of bone healing.

**Figure 3:**
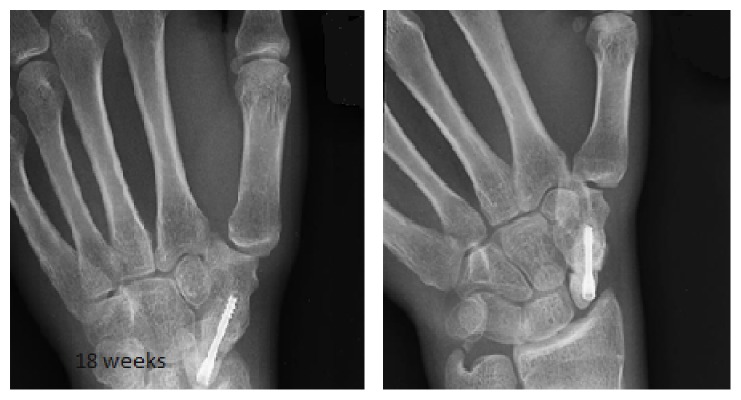
Radiographs show fracture fixation 18 weeks postoperatively.

Plaster was then removed and patient was assessed clinically and found to have some stiffness of the wrist joint but no bony tenderness over the operative site. Patient was then referred to physiotherapy and reviewed again after eight weeks and full range of motion was achieved and further X rays showed satisfactory bony union (Figure 3).

## 3. Discussion

Carpal synostoses are described in the literature as rare entities and occur in about 0.1% of the population and more common in the Afro-Caribbean and females [2, 4]. These originate from incomplete cavitation at the site of the future joint space with subsequent chondrification and ossification during the 4th to 8th weeks of intrauterine life and it may be transmitted as an autosomal dominant disorder [1, 5, 6]. Carpal synostoses are invariably asymptomatic, since almost always they are incidental findings.

Carpal synostoses can be diagnosed as an isolated anomaly or part of a congenital syndrome or can be acquired [3]. Those linking the proximal and the distal carpal rows or affecting more than one carpal bone are believed to be observed in congenital or acquired carpal bone abnormalities, while synostoses in the same row are likely to be an isolated anomaly [6, 7].

Carpal synostoses combinations have been reported between mostly all of the carpal bones with lunotriquetrum followed by capitohamate being the most frequent and Scaphotrapezial synostosis is the rarest type [8–12]. Scaphotrapezial synostoses can be associated with other syndromes like hand-foot-uterus syndrome, symphalangism, and otopalataldigital syndrome [13–15]. Carlson stated that synostosis does not work in favor of strengthening the carpus nor decreasing the chances of sustaining fractures when subjected to trauma [16].

Weathers et al. [17] have reported 3 cases of Scaphotrapezial coalition which was noted incidentally during trauma workup but none of these cases were associated with scaphoid fracture. Further, Campaigniac et al. [15] were the first to report a symptomatic unilateral Scaphotrapezial synostosis which was managed successfully with excision of the fibrous band and placement of interposition fat graft.

Park and Goddard were the first to report Scaphotrapezial coalition with concomitant scaphoid fracture in a 15-year-old Afro-Caribbean girl which was managed successfully with percutaneous scaphoid fixation using headless screw via dorsal approach [18]. In our case report, the surgeon has chosen to fix the scaphoid in an antegrade fashion through dorsal approach as volar approach could have been more challenging because of the proximity of the fracture and would have possibly required a longer screw. Because of the synostosis there was no concern with regard to penetrating the Scaphotrapezial joint as the joint demarcation does no longer exist.

## 4. Conclusion

Scaphotrapezial synostosis has been rarely reported in the literature and only one case underwent surgical treatment for scaphoid fracture. This is the second case to report a successful surgical treatment for a scaphoid fracture with a concomitant Scaphotrapezial synostosis. We fixed this fracture in an antegrade fashion using headless, self-tapping, variable-pitch compression screw through a dorsal approach. There were no perioperative complications and satisfactory bone healing was achieved in about ten weeks.
